# A software application for comparing large numbers of high resolution MALDI-FTICR MS spectra demonstrated by searching candidate biomarkers for glioma blood vessel formation

**DOI:** 10.1186/1471-2105-9-133

**Published:** 2008-03-01

**Authors:** Mark K Titulaer, Dana AN Mustafa, Ivar Siccama, Marco Konijnenburg, Peter C Burgers, Arno C Andeweg, Peter AE Sillevis Smitt, Johan M Kros, Theo M Luider

**Affiliations:** 1 Department of Neurology, Laboratory of Neuro-Oncology, Clinical and Cancer Proteomics, Erasmus Medical Center, Dr. Molewaterplein 50, P.O. Box 2040, 3000 CA Rotterdam, The Netherlands; 2 Department of Pathology, Erasmus Medical Center, Dr. Molewaterplein 50, P.O. Box 2040, 3000 CA Rotterdam, The Netherlands; 3 FOM-institute for Atomic and Molecular Physics, Kruislaan 407, 1098 SJ Amsterdam, The Netherlands; 4 Department of Virology, Erasmus Medical Center, Dr. Molewaterplein 50, P.O. Box 2040, 3000 CA Rotterdam, The Netherlands

## Abstract

**Background:**

A Java™ application is presented, which compares large numbers (n > 100) of raw FTICR mass spectra from patients and controls. Two peptide profile matrices can be produced simultaneously, one with occurrences of peptide masses in samples and another with the intensity of common peak masses in all the measured samples, using the peak- and background intensities of the raw data. In latter way, more significantly differentially expressed peptides are found between groups than just using the presence or absence in samples of common peak masses. The software application is tested by searching angiogenesis related proteins in glioma by comparing laser capture micro dissected- and enzymatic by trypsin digested tissue sections.

**Results:**

By hierarchical clustering of the presence-absence matrix, it appears that proteins, such as hemoglobin alpha and delta subunit, fibrinogen beta and gamma chain precursor, tubulin specific chaperone A, epidermal fatty acid binding protein, neutrophil gelatinase-associated lipocalin precursor, peptidyl tRNA hydrolase 2 mitochondrial precursor, placenta specific growth hormone, and zinc finger CCHC domain containing protein 13 are significantly different expressed in glioma vessels. The up-regulated proteins in the glioma vessels with respect to the normal vessels determined by the Wilcoxon-Mann-Whitney test on the intensity matrix are vimentin, glial fibrillary acidic protein, serum albumin precursor, annexin A5, alpha cardiac and beta actin, type I cytoskeletal 10 keratin, calcium binding protein p22, and desmin. Peptide masses of calcium binding protein p22, Cdc42 effector protein 3, fibronectin precursor, and myosin-9 are exclusively present in glioma vessels. Some peptide fragments of non-muscular myosin-9 at the C-terminus are strongly up-regulated in the glioma vessels with respect to the normal vessels.

**Conclusion:**

The less rigorous than in general used commercial propriety software de-isotope algorithm results in more mono-isotopic peptide masses and consequently more proteins. Centroiding of peptide masses takes place by taking the average over more spectra in the profile matrix. Cytoskeleton proteins and proteins involved in the calcium signaling pathway seem to be most up-regulated in glioma vessels. The finding that peptides at the C-terminus of myosin-9 are up-regulated could be ascribed to splicing or fragmentation by proteases.

## Background

**Figure 1:**
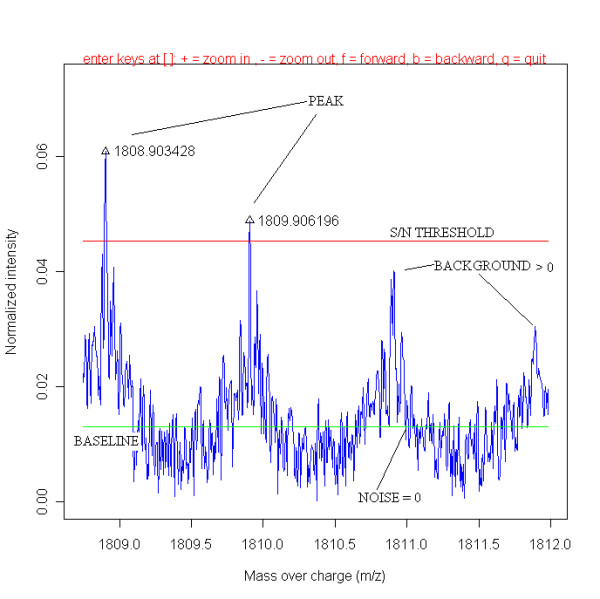
**A fragment of a MALDI-FTICR MS spectrum as generated from the raw binary fid file**. The baseline is the bottom horizontal line. The signal to noise threshold is the upper horizontal line. The peaks at masses 1808.9034 Da and 1809.9062 Da have intensities above the S/N threshold. The masses at 1808.9034 and 1809.9062 Da are used to create the peptide profile matrix. The intensities of all four peaks, including the background signal at approximately masses at 1810.9 and 1811.9 Da are used in the intensity peptide profile matrix.

Gliomas are the most common primary brain tumors, which resort among the neoplasms with the highest degree of blood vessel formation. The identification of new angiogenesis-related proteins is important for the development of anti-angiogenesis therapy. In a previous study [1], angiogenesis related proteins were identified in micro-dissected glioma vessels using Matrix Assisted Laser Desorption Ionization Fourier Transform Ion Cyclotron Resonance (MALDI-FTICR) Mass Spectrometry (MS). In brief, four different micro-dissected tissue groups were compared, namely, 1) samples from glioma blood vessels, 2) samples from glioma tissue surrounding these glioma vessels, 3) samples blood vessels in normal brain, and 4) samples from tissue that surrounded the normal vessels. The study resulted in the discovery of enzymatic by trypsin digested peptide fragments of proteins, which are exclusively present in samples of the glioma blood vessels using MS. The presence of two of these proteins was confirmed with specific antibodies on various tissue sections including glioma. The combination of techniques led to the discovery of validated biomarkers for glioma vessels, respectively, fibronectin precursor with SwissProt™ accession code P02751, collagen-binding protein 2, also named colligin-2 with SwissProt™ accession code P50454, and one candidate marker acidic calponin-3 with SwissProt™ accession code Q15417. The presence of fibronectin precursor and colligin-2 was confirmed by staining of tissue sections with specific commercial antibodies. Staining of fibronectin and colligin-2 by immunohistochemistry in glioma vessels is shown in Figure 3 and Figure 4 of ref. [1]. However, it is desirable to find more than these 3 proteins as candidate biomarkers. Ideally, the finding of more specific proteins will help to elucidate protein pathways that function in angiogenesis. Sophisticated bioinformatics may retrieve more information from present MS data. In a previous manuscript, a database application is presented, which enables to compare hundreds of MALDI-TOF MS spectra from patient and control groups [2]. In this study, the application, written in Java™ [3], is adapted to handle hundreds of mass spectra, which are measured on a FTICR MS instrument. This required complete rewriting of some parts of the source code, avoiding computer memory and performance issues dealing with high resolution FTICR MS spectra. The new architecture can also compare intensities of spectra as we address later. This was not possible in the previous version. The high resolution of MALDI FTICR spectra with respect to MALDI-TOF spectra enables de-isotoping, which we implemented in current version. In addition, the application can handle LC MALDI MS peak lists, which de-convolutes peptide masses with extra dimension elution time. Table 1 gives an overview of all the instruments and file types of exported mass spectra that are accessible for the application. The files types may consist of raw binary MS spectra or exported peak lists containing masses and corresponding intensities or XML files. The use of peak lists or XML files with respect to the use of raw binary data (the fid files) has the disadvantage of the so called "missing data" problem, which is demonstrated in Table 2a. When comparing peak lists, it appears that the signal intensity from a specific peak in a sample is not matched with intensities of peptide masses from other samples. This problem is tackled by using the raw binary data generated from the mass spectrometer instead of peak lists. The database application is adapted to handle raw binary fid (free induction decay) files. The data in these files is processed by Fast Fourier Transformation (FFT) into a frequency signal. By this conversion the mass can be calculated from the frequency, using the calibration constants in the acqus (acquisition status) files. The average signal intensity of noise, the baseline, is calculated according to a method developed by Horn et al. [4]. Figure 1 shows a fragment of an MALDI-FTICR MS spectrum as generated from the raw binary fid file. The baseline is the bottom horizontal line in Figure 1. The scatter of noise intensities around the baseline as seen in Figure 1 is expressed in a variable noise N. Real peaks are expected to display a signal, S, with an intensity above the sum of 2 intensities, namely 1) the baseline intensity, and 2) the factor*N. The combined intensity is denoted as signal to noise, S/N, threshold. The signal to noise threshold is the upper horizontal line in Figure 1. The peaks at masses 1808.9034 and 1809.9062 Da have intensities above the S/N threshold. The masses 1808.9034 and 1809.9062 Da are added in the peptide profile matrix with number of occurrences of a peptide mass for different samples [2]. Average masses over all spectra are calculated from these masses in the different samples with intensities above the S/N threshold. When working with raw data creating the intensity matrix, it is possible to register the background noise signals by recording all the peaks, such as approximately at 1810.9 and 1811.9 Da in Figure 1 with intensities between the baseline and the S/N threshold in a separate "noise peaks" file. These masses are not added to common peak list of the peptide profile matrix. Solely the background intensities > 0 but smaller than the S/N threshold of these "noise peaks" file are used for calculations. All signals below the baseline are neglected. This approach results in a more reliable and complete comparison of the intensities of peak masses in different groups (Table 2b). For each mass of the common peak list a Wilcoxon-Mann-Whitney rank sum test can be performed comparing the intensities of this mass in the samples of two groups. The p-value of the Wilcoxon-Mann-Whitney test ranks peak masses, that have for instance intensities that are strong up-regulated in the glioma vessel group compared to the normal control vessel group, and vice versa. In this way, we can find more significantly differentially expressed peptides, than just using the presence-absence matrix of peak masses. In a presence-absence matrix the presence of a specific mass in a sample, part of a common peak list of the masses in all samples, is represented by 1, and the absence is represented by 0. The process of generating the binary matrix is explained in [2] with that difference that in this FTMS study no replicate spectra of samples are measured, resulting in counted values of just 1 and 0. The peak lists of the samples are processed from raw data with high sensitivity at a relative low S/N threshold of 4. It is well known that MALDI TOF MS analysis suffers from limited reproducibility in term of peak intensities. The peak intensities measured with the FTICR MS, however, vary about 10% in replicate spectra of a single sample. The peak intensities are not normalized, because experimental conditions are kept constant, e.g. number of laser shots. A simple, but effective de-isotope algorithm is introduced, which is able to remove most of the isotopic masses from the raw data. The resulting peak list of one sample is compared with that obtained with commercially DataAnalysis™ software (Bruker Daltonics, Germany), which uses the SNAP™ de-isotope algorithm. The tryptic protonated peptide fragments of proteins are compared with those theoretically calculated from the proteins in the SwissProt™ database, using the MASCOT™ search engine [5] and MS-MS sequencing of MALDI-TOF MS data of Liquid Chromatography (LC) fractionated samples using the WARP-LC™ software (Bruker Daltonics, Germany) as described in [1].

**Table 1:** Overview of all the instruments and MS file types that can be handled by the database application.

Equipment	Type	File names	File content
Ultraflex ™ MALDI-TOF (Bruker Daltonics)	fid	fid, acqus, acqu	raw binary data, calibration constants
	text	*.txt	mass and intensity peak list, space or tab separated

APEX-Q 9.4T MALDI-FTICR (Bruker Daltonics)	fid	fid, acqus, acqu	raw binary data, calibration constants
	text	*.txt	mass and intensity peak list, space or tab separated
	xml	exportedSpectrum.xml	<pk mz="mass1" i="intensity1"/>

Off-line LC-MALDI (TOF/TOF) (file-export WARP-LC™ software, Bruker Daltonics)	text	*.txt	mass, intensity and Retention time peak list, space or tab separated data
	xml	*.xml	<Compound mass="mass1" RetentionTime="retentiontime1" AbsoluteIntensity="intensity1"> </Compound>

The database application is written in Java and R and uses a MySQL™ database. The files types may consist of 1) raw binary MS spectra (the fid files), 2) exported peak lists containing tab or space separated masses and corresponding intensities, and 3) XML files.

**Table 2:** A fragment of mass and intensity peaks lists of 2 samples, illustrating the "missing data" problem.

A)				
		
Sample 1			Sample 2	
		
Mass (Da)	Intensity (A.U.)		Mass (Da)	Intensity (A.U.)
	?	←	1193.6166	756021
	?	←	1194.5728	191048
1194.6477	175777		1194.6477	271465
1197.1376	126487	→		?
	?	←	1197.6611	124472
	?	←	1198.5193	162275
1198.7047	3154774		1198.7047	2435350
		
B)				
		
Sample 1			Sample 2	
		
Mass (Da)	Intensity (A.U.)		Mass (Da)	Intensity (A.U.)
	0	←	1193.6166	756021
	114322	←	1194.5728	191048
1194.6477	175777		1194.6477	271465
1197.1376	126487	→		80604
	0	←	1197.6611	124472
	0	←	1198.5193	162275
1198.7047	3154774		1198.7047	2435350

A) Missing data problem. It appears that the signal intensity from of a peptide mass of 1193.6166 Da in sample 2 is not matched with an intensity of the peptide mass from another sample 1 and vice versa the intensity of peptide mass of 1197.1376 Da of sample 2 is not observed in sample 1.

B) More reliable comparison including background noise signal by recording all the peaks with intensities between the baseline and the S/N threshold in a "noise peaks" file.

A.U. = arbitrary units

### Mass accuracy

In the peak picking algorithm the data point are used with the local highest intensity without an m/z centroiding. In Equation (2) to (5) the frequency and the mass difference between the data points is given to describe the inaccuracy due to this simple peak detection. We do not consider mass accuracy due to peak broadening by space charging. In our experiments, we accumulate the ions of 10 laser shots in the storage hexapole for each scan, and a total of 100 scans is used for each mass spectrum. Storing the ions in the hexapole prior to ICR analysis prevents overloading of the ICR cell. The mass accuracy in ppm of peak maxima in FTICR spectra can theoretically be calculated for the FTICR MS device used as described in any advanced MS textbook [6]. Recapitulated, the dimensionless value of $\frac{\text{m}}{\text{z}}$ is inversely proportional to the frequency f in Hz of the FTICR signal for a particle with charge z*e (C) and mass m*u (kg).

(1) $$\frac{\text{m}}{\text{z}}=\frac{\text{ML1}}{\text{f}}\approx \frac{\text{e}*\text{B}}{\left(\text{u}*2\pi *\text{f}\right)}$$

where the device specific calibration constant in the acqus file ML1 ≈ 1.443*10^8 ^Hz is used for calculating peak masses from frequencies. A derivative of above equation originates in the following equation [6]:

(2) $$\begin{array}{cc} \frac{\Delta \left(\frac{\text{m}}{\text{z}}\right)}{\frac{\text{m}}{\text{z}}}=-10^{6}*\frac{\frac{\text{m}}{\text{z}}}{\text{ML}1}*\Delta \text{f} & \text{(ppm)} \end{array}$$

The frequency resolution Δf is:

(3) $$\begin{array}{cc} \Delta \text{f}=\frac{2*{\text{SW}}_{\text{h}}}{\text{TD}*\text{zerofilling}} & \left(\text{Hz}\right) \end{array}$$

where the frequency sweep SW_h is 1.818*10^5 ^Hz; the number of data points TD = 524288 and a chosen factor zerofilling is 4. Equation (3) shows that zero filling increases the mass accuracy [7]. Applying these values results in:

(4) $$\begin{array}{cc} \frac{\Delta \left(\frac{\text{m}}{\text{z}}\right)}{\frac{\text{m}}{\text{z}}}=-1.202*10^{-3}*\frac{\text{m}}{\text{z}} & \left(\text{ppm}\right) \end{array}$$

The average distance between the real maximum and the raw spectrum data point is $\frac{1}{2}$ the distance between the data points. Therefore the absolute value of mass accuracy for a 9.4 T ICR magnet is:

(5) $$\begin{array}{cc} \text{Mass accuracy}=-\frac{1}{2}*\frac{\Delta \left(\frac{\text{m}}{\text{z}}\right)}{\frac{\text{m}}{\text{z}}}\approx 0.6*10^{-3}*\frac{\text{m}}{\text{z}} & \left(\text{ppm}\right) \end{array}$$

For a dimensionless $\frac{\text{m}}{\text{z}}$ value of for instance 1000 equal to Daltons for single charged MALDI masses, a mass accuracy of 0.6 ppm can be calculated and for a dimensionless $\frac{\text{m}}{\text{z}}$ value of 2500, a mass accuracy of 1.5 ppm can be theoretically be expected. The average value of mass accuracy for a $\frac{\text{m}}{\text{z}}$ between a 1000 and 2500 is 1.05 ± 0.27 ppm. Given these values, corresponding peptide masses of proteins where searched in the SwissProt™ database with MASCOT™ using a mass tolerance of peptides of no more than 2 ppm.

### De-isotope algorithm

A simple, but effective, de-isotope algorithm is implemented in the database application based on the methodology described earlier [8]. The de-isotope algorithm starts with the determination of a number of isotopic clusters of peaks above the signal to noise threshold (Figure 1), denoted as C, in the mass spectrum, where the difference in peptide mass between the mono-isotope ${\text{M}}_{\text{j}}^{0}$ and first isotope ${\text{M}}_{\text{j}}^{1+}$ in each cluster, j, is ${\text{mass M}}_{\text{j}}^{1+}-{\text{mass M}}_{\text{j}}^{0}$, and the difference in mass of the first and second isotope, ${\text{mass M}}_{\text{j}}^{2+}-{\text{mass M}}_{\text{j}}^{1+}$, and so on. The theoretical isotopic difference ${\text{mass M}}_{\text{j}}^{1+}-{\text{mass M}}_{\text{j}}^{0}$ reported for the first isotope in Figure 1a of ref. [4] is about 1.00289 Da. The relative atomic abundance in the theoretical amino acid "averagine" is 31.71% C, 49.82% H, 8.72% N, 9.49% O, and 0.27% S [9], while the abundance of the first isotope C^13 ^is 1.11% with an isotopic difference mass C^13 ^– C^12 ^= 1.00335 Da, 0.015% H^2 ^with 1.00628 Da, 0.370% N^15 ^with 0.99704 Da, 0.037% O^17 ^with 1.00423 Da, 0.700% S^33 ^with 0.99939 Da [10]. Based on a weighted mean, a value of 1.00289 Da can be calculated for the first isotope. Horn et al. [4] calculate the isotopic distances also for higher isotopes with an average value of 1.00235 Da. When applying an input isotopic distance of 1.00235 Da [4] in the application, the average isotopic distance in the best clusters as will be described shortly in 38 samples is 1.00327 Da. Clearly the isotopic distance in the best clusters is predominantly influenced by the C^13 ^isotopic difference of 1.0034 Da, an experimental value which we most frequently observe in our spectra. This might be due to ion statistics, because with low concentrations of sample, average values are not measured. The value 1.0034 Da is used as a default setting in the application. The intensity of the mono-isotope peak in the mass spectrum may be larger than the intensity of the first isotope in the low mass area m/z < 1800, or the intensity of the mono-isotope peak may be smaller than the first, second, or higher isotope peak when m/z > 1800 [11,12]. However, in both cases the intensity of subsequent isotopes in a cluster never increases, once it has decreased (at least not with non-overlapping isotopic clusters). Furthermore, a mono-isotope peak in this algorithm does not have an accompanying isotopic peak with an approximately 1.0034 Da smaller mass. These considerations are taken into account when determining the peptide masses that belong to true isotopic clusters. Within each isotopic cluster all peptide masses are gathered which have an m/z distance with the previous isotope of 1.0034 ± 0.0100, thus with an initial relative high mass tolerance of 1%. The average isotopic distance of each cluster j is calculated according to:

(6) $$\mu_{\text{j}}=\frac{\sum_{\text{i}=0}^{{\text{N}}_{\text{j}}-2}\left({\text{mass M}}_{\text{j}}^{(\text{i}+1)+}-{\text{mass M}}_{\text{j}}^{\text{i}+}\right)}{\left({\text{N}}_{\text{j}}-1\right)}$$

Where *N*_j _is the number of peptide masses in an isotopic cluster, j, including the mono-isotope, ${\text{M}}_{\text{j}}^{0}$. From the total amount of clusters C, a number of clusters are selected; C_s _(≤ C) that shows the smallest intra isotopic distance variance, for example less than 0.1%. For each selected cluster j:

(7) $$\sqrt{\frac{\left[\sum_{\text{i}=0}^{{\text{N}}_{\text{j}}-2}{\left({\text{mass M}}_{\text{j}}^{\left(\text{i}+1\right)}-{\text{mass M}}_{\text{j}}^{\text{i}+}\right)}^{2}\right]-\left({\text{N}}_{\text{j}}-1\right)\mu_{\text{j}}^{2}}{\left({\text{N}}_{\text{j}}-1\right)}}<\frac{{\text{variance}}_{{\text{isotopic}}_{\text{distance}}}}{100}$$

In this case the variable variance_isotopic_distance = 0.1. If at least 10 good clusters are found, C_s _≥ 10, a new average isotopic distance, *μ*_s_, is calculated from the mean isotopic distance, *μ*_j_, of the selected clusters, j.

(8) $$\mu_{\text{s}}=\frac{\sum_{\text{j}=0}^{{\text{C}}_{\text{s}}-1}\mu_{\text{j}}}{{\text{C}}_{\text{s}}}$$

The mean isotopic distance *μ*_s _of the selected clusters has a deviation of:

(9) $$\sigma_{\text{s}}=\sqrt{\frac{\left[\sum_{\text{j}=0}^{{\text{C}}_{\text{s}}}\mu_{\text{j}}^{2}\right]-\left({\text{C}}_{\text{s}}*\mu_{\text{s}}^{2}\right)}{{\text{C}}_{\text{s}}-1}}$$

**Figure 2:**
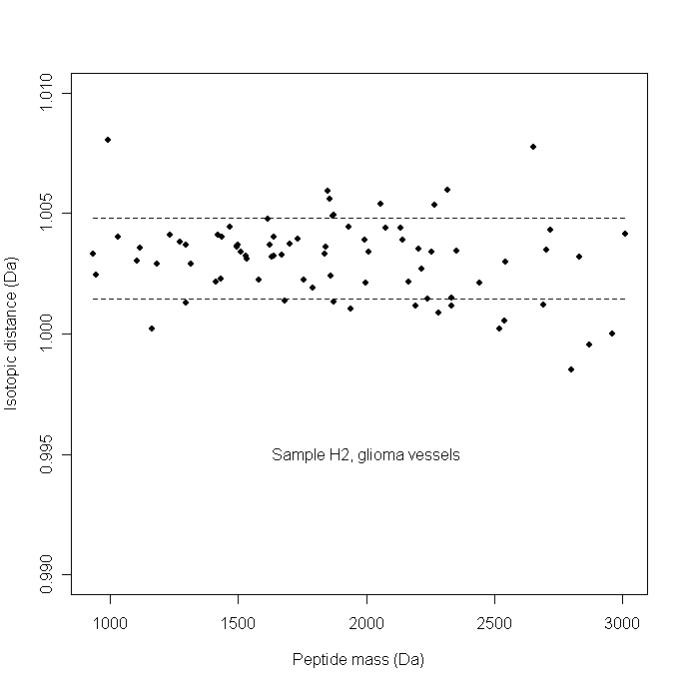
**Distribution of the mean isotopic distances *μ*_j _of real isotopic clusters as a function of the mono-isotopic peptide mass in the glioma vessels sample H2**. The dashed lines represent the acceptance window, between *μ*_s _– *σ*_s _and *μ*_s _+ *σ*_s _of mean isotopic distances of real isotopic clusters. The larger mass accuracy with larger peptide masses is reflected by the larger scatter of isotopic distances *μ*_j_

True isotopic clusters must have a mean isotopic distance, *μ*_j_, between *μ*_s _– *σ*_s _and *μ*_s _+ *σ*_s_. Figure 2 shows the distribution of the mean isotopic distance, *μ*_j _of potential isotopic clusters of sample H2 as a function of the mono-isotopic masses. The dashed line in Figure 2 represents the acceptance window of mean isotopic distances real clusters, *μ*_s _– *σ*_s _and *μ*_s _+ *σ*_s. _As shown in Figure 2, the larger mass accuracy at larger peptide masses is reflected by the larger scatter of isotopic distances *μ*_j_. All isotopic masses of the clusters, that have a mean isotopic distance within the acceptance window, *μ*_s _– *σ*_s _and *μ*_s _+ *σ*_s_, are removed from the peak lists. If the mean isotopic distance value of a potential isotopic cluster in not in the acceptance window, isotopic masses are peeled from this cluster from high to low mass until the mean isotopic mass is within the acceptance window, the remaining isotopes are removed from the peak list. As an example, the masses 1300.529351, 1301.531681, and 1302.525368 Da are detected as a potential isotopic cluster. However, the mean isotopic distance is too low with 0.9980 Da, due to the low distance between the first and second isotope of 0.9937 Da. The peak at mass 1302.525368 Da appears not an isotopic signal after visual inspection of the mass spectrum. Consequently, only the isotopic mass 1301.531681 Da with distance 1.0023 Da from the mono-isotope is removed from the peak list.

## Methods

### Tissue Sections

Identical raw MS fid files of 40 micro-dissected tissue sections were used as described in [1]. The 10 spectra of glioma blood vessels were coded H1 to H10, 10 spectra of tissue surrounding the glioma vessels were coded TH1 to TH10, 10 spectra of normal vessels were coded S1 to S10, and 10 spectra of tissue surrounding the normal vessels were coded TS1 to TS10.

### MS-MS sequencing of peptides in tissue sections

Proteins were identified by the MS-MS sequencing as described in [1]. In brief, a number of 4 pooled samples were subjected to nano-LC fractionation. First, 8 sections comprising tissue and vessels of sample TH8 were combined (of which 10% was estimated to be vessels). Secondly, for comparison 8 sections of the normal brain sample TS5 were combined in exactly the same way. In addition, micro-dissected glioma samples of series H1 to H10 were combined, resulting in one pooled glioma blood vessel sample. Finally, the 10 samples of normal vessels in series S1 to S10 were pooled according to the same procedure. With time intervals of 15 s, fractions of the samples were spotted automatically upon a 384 pre-spotted anchor-chip plate. The plates were measured by an automated Ultraflex™ MALDI TOF-TOF instrument (Bruker Daltonics, Germany), using WARLP-LC™ software. The WARLP-LC™ software interprets MS spectra of each individual spot and subsequently performs MS-MS on each peptide peak mass. The best peak masses for performing the MS-MS sequencing were determined automatically by the WARLP-LC™ software. The BTDX.xml export files, containing the MS and the MS-MS peak masses, were imported in Biotools™ software version 3.0 (build 1.68) (Bruker Daltonics, Germany) and submitted by this software application to the SwissProt™ version 40.21 database, using the MASCOT™ search engine. A 150 ppm parent mass tolerance, 0.5 Da fragments tolerance, and one possible missed trypsin cleavage site was allowed.

### MALDI-FTICR MS measurements

The samples of H1 toH10, S1 to S10, TH1 to TH10, and TS1 to TS10 were enzymatic digested by trypsin, mixed with 2,5-dihydroxybenzoic acid (DHB) solution (1 mg/ml H_2_O), spotted upon a 600/384 anchor-chip plate (Bruker Daltonics, Germany), and measured by an type APEX-Q™ FTICR MS instrument with a 9.4 T magnet (Bruker Daltonics, Germany). The details of this procedure is described in [1]. The mono-isotopic peak list of glioma sample H7 obtained with the new de-isotope algorithm of the database application was compared with that obtained by the SNAP™ algorithm using DataAnalysis™ version 3.4 (Build 169) software using an S/N > 1.7. The SNAP™ de-isotope algorithm is performed with the following parameters; the instrument type is set default (Fourier transform), the quality factor threshold 0, the S/N threshold 1.7, the relative intensity threshold (base peak) 0.01%, the absolute intensity threshold 0, the maximum charge state 4, the repetitive building block of C 4.9384 N 1.3577 O 1.4773 S 0.0417 H 7.7583 (the theoretical "averagine" amino acid composition [13]), the additional constant unit "empty", the algorithm version 2.0, an include component isotope pattern checkbox "not checked", the filter exclusion masses checkbox "not checked", the use peak finder to calculate peak position checkbox "checked".

### Data analysis

The Java™ software package described in [2] was adapted to handle raw FTICR MS data and was used to create a profile matrix of all peptide masses present in the 40 samples from the MALDI-FTICR mass spectra. The application was adapted to annotate peptide peak masses from raw FTICR MS fid files (Bruker Daltonics, Germany), which had intensities above an S/N threshold of 4. The search window for peaks was 20 ppm in both directions, which could be varied in the Graphical User Interface (GUI) of the application [2]. A window of 20 ppm was chosen, because of the experimental constraints of the simple peak picking (no interpolation between data points or curve fittings). The peak picking algorithm searches for a local maximum in this window in the MALDI-FTICR MS spectrum. A too small window of 3 ppm generates too large peak lists and causes performance problems. The software package was written in Java™ and R [14] and used a MySQL™ database [15]. It required special libraries to be installed, namely edtftpj-1.4.8.jar, mysql-connector-java-3.1.6-bin.jar and serializer.jar [15-17].

### Apodization Function

The following apodization function F_k _was multiplied with the raw FTICR time signal of each data point, k, before applying the FFT:

(10) $${\text{F}}_{\text{k}}={\text{e}}^{\left(-\pi *\text{LB}*\frac{\text{k}}{\text{TD}}\right)+\left(-\pi *\text{GB}*{\left(\frac{\text{k}}{\text{TD}}\right)}^{2}\right)}$$

Where the number of data points TD = 524288 (with no factor for zero filling) and 1 ≤ k ≤ TD. LB and GB represent the Lorentzian and Gaussian broadening factors. The spectra were processed with a LB of 0 and a GB of 0.3276932. The values of these factors are default settings of the application GUI.

### Internal calibration

All mass spectra of the samples were internally calibrated on ubiquitous cytoplasmic 1 beta actin single charged peptide masses of 1198.70545, 1515.74913, 1790.89186, 2215.06990 and 3183.61423 Da (see additional file 1). The protein cytoplasmic 1 beta actin has the SwissProt™ accession code P60709. The internal calibration required that measured actin masses had to be within 30 ppm distance of the calibration masses with allowance of one missing actin mass, resulting in at least 4 calibration points for each calibrated spectrum. A shift of 30 ppm was chosen since this value is somewhat smaller than the broadness of peaks at the baseline in the FTICR MS spectra with our settings and larger than the expected shift of mass values. A too small value < 5 ppm would result in a to small amount calibration masses and result in excluding the spectrum. A too large value > 30 ppm would result in the usage of peptide masses from wrong proteins other than actin. The shift by internal calibration is smaller than 5 ppm. The value of 30 ppm can be changed in the GUI of the application. An insufficient number of calibrate masses were found with the "normal vessel" sample S5 and "tissue surrounding the normal vessels" sample TS5 and they were not used for further analysis. If more than three calibration masses are found within 30 ppm of the measured peak masses, the following quadratic equation is applied to calculate the constants a [0], a[1], and a[2] from the observed frequencies of the peak masses, using R's linear model lm [14].

(11) $${\text{m}}_{\text{calibrant}}=\frac{\text{a}[1]}{{\text{f}}_{\text{measured for calibrant}}^{2}}+\frac{\text{a}[2]}{{\text{f}}_{\text{measured for calibrant}}}+\text{a}[0]$$

All peak masses were subsequently recalculated from the observed frequencies using this formula.

### Peptide profiles

Two different peptide profile matrices were produced simultaneously, one with the presence or absence of common peptide masses in spectra of different samples, and a matrix with the intensity of the peak mass if present in the sample or the intensity of the background signal if absent [2]. To avoid including too many noise peaks in the matrix, each peptide mass had to be present 10% of the spectra (= 4). The binary presence-absence matrix was transposed in Spotfire™ Decision Site 9 (SP1) version 17.2.783 [18] and an unsupervised hierarchical clustering in two dimensions was performed. The clustering was done in a number of steps, first transposing the peptide profile matrix, clustering the samples and sorting on hierarchical clustering id, than transposing the table again, and clustering the masses the second time. The clustering method used in Spotfire™ was the Un-weighted Pair Group Method with Arithmetic Mean (UPGMA) using the Euclidean distance and ordering function average value. The added column name was Hierarchical clustering and the checkbox calculate dendrogram checked. On the intensity matrix [2], a Wilcoxon-Mann-Whitney rank sum test was performed. The Wilcoxon-Mann-Whitney p-values for peptide masses were calculated for their difference in peak intensities in the normal and glioma blood vessels group. The p-values of the Wilcoxon-Mann-Whitney test on the glioma and normal vessel group are given in Additional file 2. The most up-regulated peptide masses in the glioma vessels with the lowest p-values were 1) compared with masses of the MS-MS sequencing table in the additional material (Additional file 1), and 2) used in MASCOT™ to search calculated peptides of proteins with a mass tolerance of 2 ppm difference with the experimental values. Two analysis rounds were performed, respectively with peptides masses that had p-values < 0.01 and p-values < 0.1.

### Mass accuracy

The experimental mass accuracy was determined from the mean value of the absolute differences between the measured and theoretical masses of tryptic peptide fragments of 2 proteins; 1) GFAP with entry name GFAP_HUMAN and SwissProt™ accession number P14136, and 2) type I cytoskeletal 10 keratin, with entry name K1C10_HUMAN and SwissProt™ accession number P13645. The protein K1C10_HUMAN was determined by the WARP-LC™ software to be present in 2 pooled samples; 1) glioma vessels, and 2) normal vessels (see Additional file 1). The protein GFAP_HUMAN was determined by the WARP-LC™ software to be present in all the 4 pooled samples; 1) glioma vessels 2) glioma tissue and vessels, 3) normal vessels, and 4) normal tissue and vessels (see Additional file 1). Keratin and Glial Fibrillary Acidic Protein were selected, since the tryptic peptide masses from these proteins appeared to be present in all the MALDI-FTICR MS spectra. From in total about 60 peak masses, the mass accuracy was determined from 20 peak masses that had the highest mean peak intensities, 20 peak masses with middle mean peak intensities, and 20 peak masses with the lowest mean peak intensities. The mean peak intensity of each mass was calculated from peak and background intensities from the present signals in the intensity matrix.

## Results

### MS-MS peptide sequencing of tissue sections

All MS-MS sequencing analysis results of 1) the glioma vessels samples, 2) glioma tissue and vessels samples, 3) normal vessels samples, and 4) normal tissue and vessels samples generated by the WARP-LC™ software are presented in the additional material (Additional file 1).

### Peak finding and de-isotope algorithm

The peak finding algorithm in our database application results in a peak list of 2026 peptide peak masses with a S/N threshold of 4 for sample H7 (Additional file 3). The DataAnalysis™ 3.4 (Build 169) software gives approximately the same number of 2064 peak masses (≈ 2026) at a S/N threshold of 1.7 (Additional file 4). The definition of S/N threshold for peak finding is different in both software applications. The overlap of both peak lists is about 90% with 1834 peptide masses. A large decrease with 82% to 379 peak masses is assigned when the SNAP™ de-isotope algorithm is applied on the peak list exported from the DataAnalysis™ software (Additional file 5). A more modest decrease with 28% to 1451 peak masses is seen with the de-isotope algorithm described in this publication (Additional file 6). A number of about 250 isotopic clusters fall within the acceptance window (Figure 2), with an average of 3 isotopes (including the mono-isotope). When about 500 isotopes are remove from the peak list (≈ 2026 – 1451), it appear that the main amount of the 1451 peaks, namely ± 1200 (≈ 1451 – 250) are single peaks with intensities just above the signal to noise threshold. A percentage of 83% (314 peak masses) of the DataAnalysis™ mono-isotopic peak masses appears to be present in the mono-isotopic list of the database application. At a higher S/N ratio of 4 instead of 1.7, using the DataAnalysis™ software, the number of total annotated peak masses gradually drops to 654, while the number of mono-isotopic peaks established with the SNAP™ algorithm yields 283 peak masses. This is a smaller decrease of 57% than the 82% measured at a low signal to noise threshold of 1.7. It indicates that the mono-isotopic peaks that are detected with the SNAP™ algorithm are in the high intensity peptide clusters.

### Mass accuracy

In Table 3, the measured mass accuracy of an exported peak list of a single sample H7 from the database application is about 1.10 ± 0.91 ppm. This mass accuracy is about equal to the theoretical value of 1.05 ± 0.27 ppm. The masses in the peak list of the same sample exported with the DataAnalysis™ (Bruker Daltonics, Germany) software are more accurate with a low value of 0.81 ± 0.59 ppm. The higher accuracy is probably due to another internal calibration formula and a sophisticated propriety centroiding algorithm. When taking into account the average of the peptide masses in all the samples, the measured mass accuracy in the database application from raw data decreases from 1.10 ± 0.91 to 1.03 ± 0.72 and further to 0.67 ± 0.48 ppm when determined on peptide masses, which appear to be presents in at least 5 or more and 18 or more samples in the peptide matrix of in total 38 samples, respectively. The peak masses with the highest intensities do not necessary have a higher mass accuracy, namely 0.91 ± 0.58 ppm, than peak masses with middle mean peak intensities, 0.66 ± 0.39 ppm. Peak masses with the lowest mean peak intensities have the worst mass accuracy, 1.54 ± 0.82 ppm, but are also calculated with the smallest number of 254 peak and background signals.

**Table 3:** Mass accuracy of peptide masses when measured with FTICR MS.

	Average mass accuracy in ppm from calculated masses	Standard deviation in ppm
Theoretical with zero-filling factor 4	1.05	0.27
Data analysis 3.4 (Build 169) peak-list of sample H7 masses with intensities higher than a threshold of signal to noise 1.7	0.81	0.59
Peak list of sample H7 exported from this application with masses with intensities higher than a threshold of signal to noise 4	1.10	0.91
Masses in the peptide profile matrix which are present in 5 or more samples of total 38 samples	1.03	0.72
Masses in the peptide profile matrix, which are present in 18 samples or more of total 38 samples	0.67	0.48

20 peak masses with the highest mean peak intensities based on 586 peak and background signals	0.91	0.58
20 peak masses with middle mean peak intensities based on 383 peak and background signals	0.66	0.39
20 peak masses with the lowest mean peak intensities based on 254 peak and background signals	1.54	0.82

Measured on Glial fibrillary acidic protein (GFAP) with SwissProt™ accession code : P14136, masses MH^+^: 856.4523, 906.46795, 911.45811, 986.52653, 988.5058, 1032.52076, 1039.55307, 1042.60036, 1050.52143, 1087.5742, 1098.62654, 1103.53273, 1108.47613, 1117.57353, 1177.62113, 1208.59055, 1215.63277, 1244.65934, 1245.66846, 1263.63274,1277.70995, 1357.75461, 1405.80492, 1409.74227, 1499.78523, 1505.76281, 1629.93224, 1634.76897, 1697.79852, 1746.91329, 1841.97153, 1853.89963, 1878.96679, 2075.14949, 2332.9597, 2348.14766 Da		

Measured on Keratin, type I cytoskeletal 10 with SwissProt™ accession code: P13645, masses MH^+ ^: 847.45197, 995.51965, 1031.59829, 1109.48979, 1118.50858, 1165.58475, 1201.61713, 1234.67896, 1237.58476, 1262.59711, 1324.61679, 1365.6393, 1381.6481, 1390.68082, 1434.76995, 1493.73429, 1707.77202, 1797.01158, 1996.97095, 2025.94334, 2096.04671, 2224.14169, 2295.12852, 2367.26265, 2872.39273, 3052.62729 Da.		

The mass accuracy is determined on tryptic fragments. The experimental mass accuracy was determined from the calculated value of tryptic peptide fragments of 2 proteins; 1) Glial fibrillary acidic protein with entry name GFAP_HUMAN and SwissProt™ accession number P14136, and 2) Keratin, type I cytoskeletal 10 with entry name K1C10_HUMAN and SwissProt™ accession number P13645.

### Hierarchical clustering

**Figure 3:**
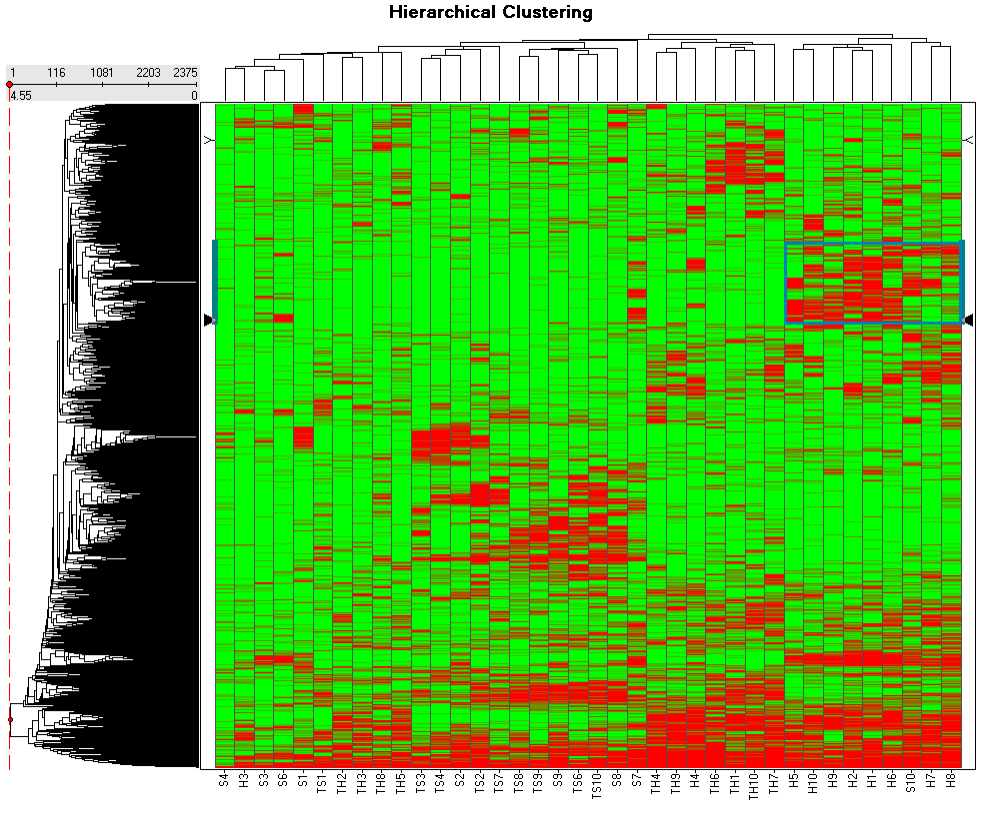
**Heat-map of the hierarchical clustering on the presence-absence peptide mass profile matrix in two dimensions of the 38 samples and 2375 peptide masses**. The data of the clustering table is added as an additional file 7. A number of 10 spectra of glioma blood vessels with codes H1 to H10, 10 spectra of tissue surrounding the glioma vessels with codes TH1 to TH10, 10 spectra of normal vessels with codes S1 to S10, and 10 spectra of tissue surrounding the normal vessels with codes TS1 to TS10 are included. Two "normal vessels" samples, S5 and TS5, were excluded because they could not be calibrated. The highlighted box in Figure 3 represents the hierarchical clustering order 490 with mass 1037.5355 Da to 789 with mass 1665.7891 Da as presented  (see Additional file 7).

Figure 3 shows the hierarchical clustering on the presence-absence peptide mass profile matrix of 38 of the in total 40 samples in a heat-map. Peptide masses present in glioma vessels clustering visually together in the red heated area in the marked blue box. This helps selecting the peptide masses to search for proteins in the SwissProt™ database using the MASCOT™ search engine. The clustering table is added as the Additional file 7. Two samples, S5 and TS5, were excluded because they could not be calibrated. Sample S10 appears to be an outlier in Figure 3, since it is clustered between samples H6 and H7 and other glioma samples. Peptide masses clustering to glioma vessels, illustrated in the highlighted box of Figure 3 are submitted to the SwissProt™ database (version 40.21, entry homo sapiens) using the MASCOT™ search engine to identify the proteins differentially expressed in the "glioma vessels" samples. A peptide mass error tolerance of ± 2 ppm is used with the MASCOT™ search. The highlighted box in Figure 3 represents the hierarchical clustering order 490 with mass 1037.5355 Da to 789 with mass 1665.7891 Da (see Additional file 7). Only proteins that have at least two matched values of experimental and calculated peptide masses are taken into consideration. One of the top 20 proteins reported by MASCOT™ is hemoglobin alpha subunit with SwissProt™ accession code P69905 with 2 matched peptide masses. Hemoglobin alpha subunit is identified by the MS-MS sequencing options of the WARP-LC™ software in the glioma vessels (Additional file 1). Hemoglobin delta subunit with SwissProt™ accession code P02042 is also found is with 2 matched peptide masses. It is identified by the MS-MS sequencing options of the WARP-LC™ software in the glioma tissue and vessels (Additional file 1). Other proteins found by the MASCOT™ search engine are Fibrinogen beta chain precursor with SwissProt™ accession code P02675 with 8 matched peptides masses and Fibrinogen gamma chain precursor with SwissProt™ accession code P02679 with 4 matched peptide masses, both identified by MS-MS (Additional file 1). The finding of hemoglobin and fibrinogen is expected, since blood proteins and clotting proteins are present in the lumina of the relative larger vessels as compared to normal vessels. Other proteins in the highlighted box found with the MASCOT™ search engine with 2 matched peptides are Tubulin-specific chaperone A with SwissProt™ accession code O75347, Fatty acid-binding protein, epidermal (E-FABP) with SwissProt™ accession code Q01469 (B-FABP is analyzed in glioma tissue and vessels, see Additional file 1), Neutrophil gelatinase-associated lipocalin precursor (NGAL) with SwissProt™ accession code P80188, Peptidyl-tRNA hydrolase 2, mitochondrial precursor with SwissProt™ accession code Q9Y3E5, Growth hormone variant precursor (GH-V) (Placenta-specific growth hormone) with SwissProt™ accession code P01242 and Zinc finger CCHC domain containing protein 13 with SwissProt™ accession code Q8WW36.

### Wilcoxon-Mann-Whitney

**Figure 4:**
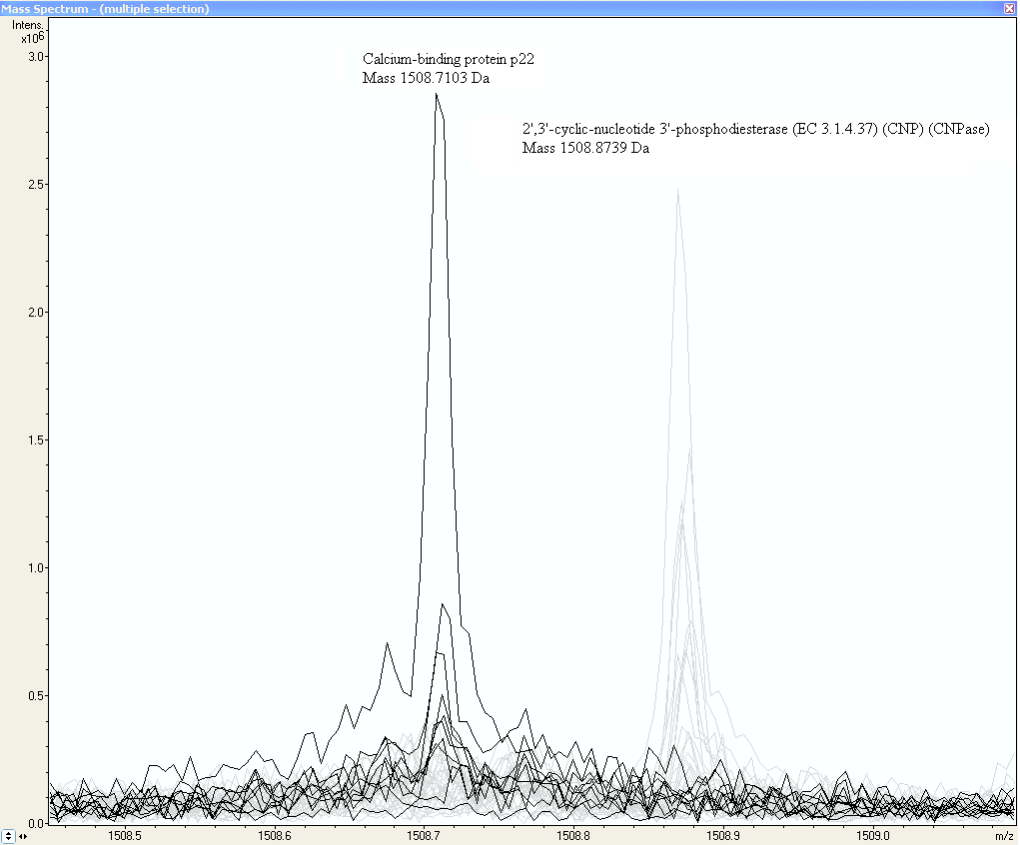
**Increased peak intensity at the mass MH^+ ^1508.7103 Da from a peptide of probably Calcium-binding protein p22 in glioma vessel samples**. The MALDI-FTICR Calcium-binding protein p22 peptide peaks of glioma samples are represented by the dark lines (by contrast the peptide mass of 2',3'-cyclic-nucleotide 3'-phosphodiesterase at 1508.8739 Da represented by the grey lines is as expected not present in glioma vessels).

The list of p-values based on the peak intensity differences between the normal and glioma vessel sample group for 2375 peptide masses is presented in the Additional file 2. A number of 95 (4%) in glioma vessels up-regulated peptide masses with p-values < 0.01 are used in MASCOT™ to search for calculated peptides masses of proteins. A mass tolerance of 2 ppm is used. The MASCOT™ search is repeated with another number of 442 (19%) in glioma vessels up-regulated peptide masses with p-values < 0.1. Table 4 summarizes the MASCOT™ search and MS-MS sequencing results of the differentially expressed proteins. Only proteins are listed that have at least three experimental measured peptide masses that correspond with the calculated values of these proteins. In Table 4, Calcium-binding protein p22 is one of the proteins with 3 peptides with a p-value < 0.1 that match with the SwissProt™ database. An increased intensity of a peak at peptide mass MH^+ ^of 1508.7103 Da of likely a peptide of Calcium-binding protein p22 is measured in glioma vessel samples represented by the dark lines in Figure 4, which shows a fraction of the MALDI-FTICR mass spectra of all samples. By contrast the peptide mass of 2',3'-cyclic-nucleotide 3'-phosphodiesterase (CNPase) at 1508.8739 Da represented by the grey lines is as expected not present in glioma vessels. This protein is identified by MS-MS sequencing in normal tissue and vessels (see Additional file 1).

**Table 4:** Proteins significantly up-regulated in the "glioma vessels" group with respect to the "normal vessels" group.

Name	SwissProt™ acc. code	Nr. hits p-value< 0.01	Nr. hits p-value< 0.1	MS-MS glioma vessels	MS-MS glioma tissue and vessels	MS-MS normal vessels	MS-MS normal tissue and vessels
Vimentin	P08670	12	18	yes	yes	no	no
Glial fibrillary acidic protein (GFAP)	P14136	3	12	yes	yes	yes	yes
Serum albumin precursor	P02768	4	10	yes	yes	no	no
Annexin A5	P08758	2	5	no	yes	no	no
Actin, cytoplasmic 1 (Beta-actin)	P60709	0	12	yes	yes	yes	yes
Keratin, type I cytoskeletal 10	P13645	0	8	yes	no	yes	no
Actin, alpha cardiac (Alpha-cardiac actin)	P68032	0	7	no	yes	no	yes
Desmin	P17661	0	6	no	yes	no	no
Calcium-binding protein p22 (Calcium-binding protein CHP)	Q99653	0	3	no	no	no	no

A number of 95 (4%) of the measured peptide masses with Wilcoxon-Mann-Whitney p-values < 0.01 and raised intensities in the glioma vessel group are compared with calculated peptide masses from proteins in the SwissProt™ database using MASCOT™. A mass tolerance of 2 ppm is used. The MASCOT™ search is repeated with another number of 442 (19%) of peptide masses that have a p-value < 0.1 and with up-regulated intensities in the "glioma vessels" group. Only proteins are listed that have at least three hits of experimental measured peptide masses corresponding within 2 ppm with the calculated peptide masses of these proteins.

### Peptide masses exclusively present in glioma vessels

The peptide masses which are exclusively present in the glioma vessels group (series H in Additional file 8) are used to search calculated masses of proteins using MASCOT™ with a mass tolerance of 2 ppm. In Table 5, all proteins are listed that have 2 hits or more by a MASCOT™ search and one of these matched peptide masses has a Wilcoxon-Mann-Whitney p-value < 0.01. MASCOT™ lists proteins due with a score. However, peptides masses can be ascribed to more than one protein in this summary. Peptide masses are preferably ascribed to proteins present in the MS-MS runs (Additional file 1) than proteins with the highest score in the MASCOT™ summary. Calcium-binding protein p22 with SwissProt™ accession number Q99653 which was found with the Wilcoxon-Mann-Whitney test is also added to Table 5. From the proteins listed in Table 5, Fibronectin precursor and Myosin-9 are also identified with the MS-MS sequencing of the pooled samples (Additional file 1), while Cdc42 effector protein 3 and Calcium-binding protein p22 are not identified by MS-MS sequencing. Except for a small peak in the outlier sample S10, Collagen-binding protein 2 precursor (Colligin-2) with SwissProt™ accession code P50454 is exclusively measured in the glioma vessels group (series H) using the new algorithm. Collagen-binding protein 2 precursor (Colligin-2) is listed in a separate Table 6. The peak intensities of the glioma samples are just above 300*10^3 ^A.U. and the value of S10 just under this value (Additional file 2).

**Table 5:** Proteins associated with glioma vessel formation established from enzymatic digested by trypsin peptide masses that are exclusively present in the "glioma vessels" group.

Measured mass MH+ (Da)	Calculated mass MH^+ ^(Da)	ppm	p-value Intensity H against S	Intensity H > S	Glioma vessels (H)	Normal vessels (S)	Glioma tissue(TH)	Normal tissue (TS)
**SwissProt^TM^ accession code Q9UKI2, name: Cdc42 effector protein 3 (Binder of Rho GTPases 2)**								
1229.6644	1229.66365	0.61	0.00411	yes	6	0	0	0
1656.8022	1656.80295	0.45	0.13120	yes	5	1^&^	0	0
1620.7609	1620.76073	0.10	0.25509	yes	4	0	0	0
1772.8870	1772.88871	0.96	0.80459	no	3	3	1	1
**SwissProt^TM^ accession code, name: Fibronectin precursor (FN) (Cold-insoluble globulin) (CIG)**								
1807.9063	1807.90469	0.89	0.00089	yes	8	0	0	0
1355.6954	1355.69536	0.03	0.00125	yes	7	0	0	0
1625.8539	1625.85196	1.19	0.00232	yes	5	0	0	0
1349.6848^*^	1349.68481	0.01	0.00345	yes	7	0	0	0
1926.0516	1926.04832	1.70	0.00503	yes	8	1^&^	0	0
1629.8728^*^	1629.87067	1.31	0.00873	yes	5	0	0	0
2470.3200	2470.31874	0.51	0.01233	yes	7	1	1	0
1078.5537	1078.55275	0.88	0.01835	no	0	3	1	1
1818.9739	1818.97078	1.72	0.02049	yes	5	0	0	0
2524.3649	2524.36568	0.31	0.02049	yes	5	0	0	0
1401.6649^*^	1401.66580	0.64	0.02170	yes	6	0	0	0
1341.6739	1341.67570	1.34	0.02663	yes	5	1	3	0
1110.5413	1110.54257	1.14	0.06243	yes	4	0	2	0
1593.8126	1593.81186	0.46	0.06243	yes	6	1	1	0
1292.6740	1292.67324	0.59	0.06373	yes	5	1	0	1
1297.7117	1297.71102	0.52	0.09473	yes	3	0	1	1
1591.8091	1591.80744	1.04	0.12668	yes	4	0	2	0
1235.5266	1235.52620	0.32	0.13120	yes	3	1	0	1
1431.7507	1431.74915	1.08	0.18754	yes	5	2	0	0
2139.9619	2139.96514	1.51	0.45543	yes	5	1	6	0
858.4301	858.43157	1.71	0.95436	no	2	1	2	0
1291.7265	1291.72560	0.70	1.00000	yes	3	3	1	0
**SwissProt^TM^ accession code Q99653, name: Calcium-binding protein p22 (Calcium-binding protein CHP) (Calcineurin homologous protein)**								
960.5588	960.55850	0.31	0.02082	yes	6	0	4	0
1138.5646	1138.56262	1.74	0.00959	yes	5	0	0	1
1508.7103	1508.70876	1.02	0.05363	yes	5	0	0	0
**SwissProt^TM^ accession code P35579, name: Myosin-9 (Myosin heavy chain, non-muscle IIa)**								
1869.9677^*^	1869.96645	0.67	0.00170	yes	6	0	0	0
1155.6643	1155.66325	0.91	0.00310	yes	5	0	0	0
1949.9955	1949.99268	1.45	0.01285	yes	8	0	0	0
2493.1740^*^	2493.17392	0.03	0.04085	yes	6	0	0	0
2472.1699	2472.17356	1.48	0.05363	yes	5	0	0	0
1393.7099^#^	1393.71102	0.80	0.07335	no	1	4	3	7
1946.0059	1946.00765	0.90	0.33559	yes	7	4	6	0
924.4946	924.49377	0.90	0.60387	yes	3	2	3	0
1309.6023^†^	1309.60188	0.32	0.61715	yes	4	3	1	0
1193.6166^*^	1193.61606	0.45	0.75200	yes	3	2	4	2
1310.7038^$^	1310.70626	1.88	1.00000	yes	1	0	7	0

The listed proteins are found by a MASCOT™ search with 2 or more hits between measured and calculated tryptic peptide masses with an error tolerance of 2 ppm. Only proteins are listed that had at least one peptide mass with a Wilcoxon-Mann-Whitney p-value < 0.01 comparing the peak intensities of glioma vessels with normal vessels.

^*^ WARP-LC MS-MS MALDI-TOF glioma tissue and vessels (see Additional file 1).

^#^ SwissProt^TM^ accession code:  P40925, name: Malate dehydrogenase, cytoplasmic, identified by WARP-LC MS-MS on glioma tissue and vessels and normal tissue and vessels (see Additional file 1).

^†^  SwissProt^TM^ accession code: P08670, name: Vimentin, sequence: NLQEAEEWYK, MALDI TOF MS-MS by hand on the “glioma tissue and vessels” LC-MALDI plate using the BiotoolsTM 3.0 (build 1.68)  software.

^$^ SwissProt^TM^ accession code:  P62805, name: Histone H4, identified by WARP-LC MS-MS on glioma tissue and vessels and normal tissue and vessels (see Additional file 1).

^&^ outlier sample S10.

**Table 6:** Distribution of tryptic peptide fragments of colligin-2 among the tissue sections of different groups Peptide masses obtained by MALDI-FTICR MS are listed that match within 2 ppm with the calculated values of enzymatic by trypsin digested fragments.

SwissProt™ accession code P50454, name: Collagen-binding protein 2 precursor (Colligin 2)								
Measured mass MH+ (Da)	Calculated mass MH^+ ^(Da)	ppm	p-value Intensity H against S	Intensity H > S	Glioma vessels (H)	Normal vessels (S)	Glioma tissue (TH)	Normal tissue (TS)
1021.5304 #	1021.53213	1.69	0.77475	no	1	2	0	3
1293.6805	1293.67973	0.60	0.01244	yes	5	1	1	3
1659.8011 *	1659.80126	0.10	0.02170	yes	5	1^&^	0	0
1675.7747 ?	1675.77577	0.64	0.01088	yes	1	0	2	4

* WARP-LC MS-MS MALDI-TOF glioma tissue and vessels (see additional file 1)

# SwissProt™ accession code: P07196, name: Neurofilament triplet L protein, identified by WARP-LC MS-MS MALDI-TOF on normal tissue and vessels (see additional file 1)

? SwissProt™ accession code: P17677, name: Neuromodulin (Axonal membrane protein GAP-43) ?

^&^outlier sample S10

### Myosin-9

**Figure 5:**
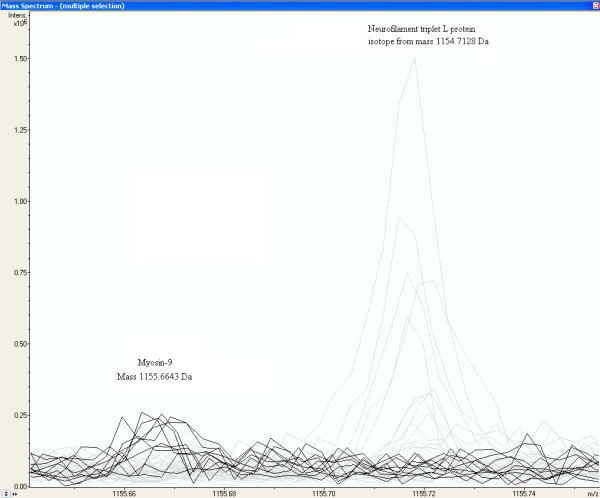
**Increased peak intensity at the mass MH^+ ^1155.6643 Da from a peptide of Myosin-9 in glioma vessel samples**. The peaks of glioma vessel samples are represented by the dark lines in MALDI-FTICR mass spectra of all samples. The first isotopic mass of Neurofilament triplet L protein at 1154.7128 Da, represented by the grey lines, is as expected not present in Glioma vessels.

**Figure 6:**
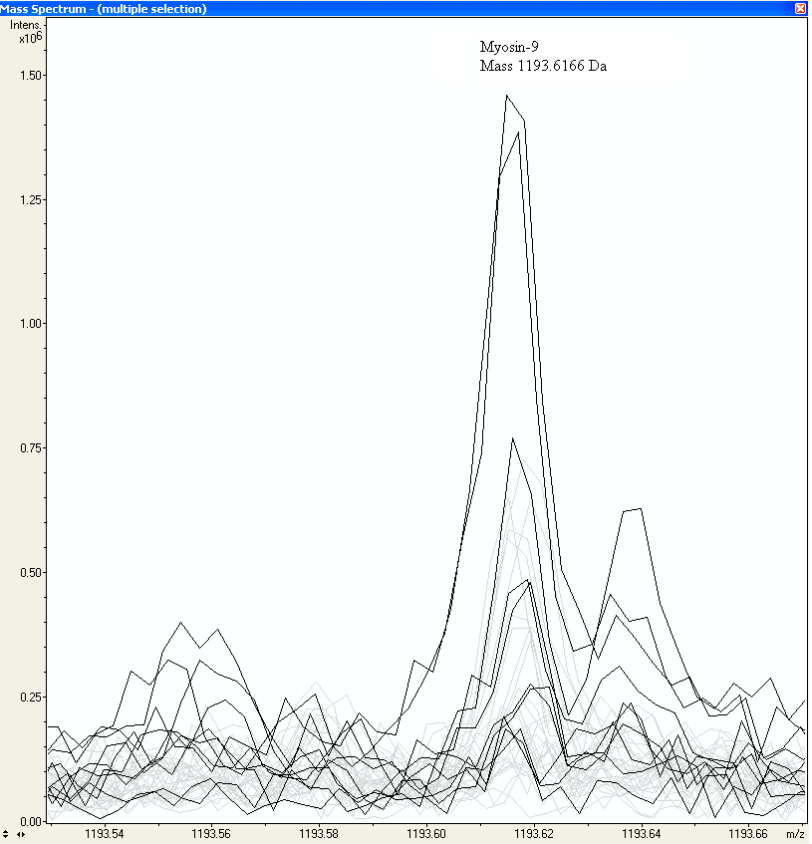
**An equal distribution of peak intensities among all samples of a Myosin-9 peptide mass at 1193.6166 Da**. The dark lines represent the peak intensities of the glioma vessel samples.

**Figure 7:**
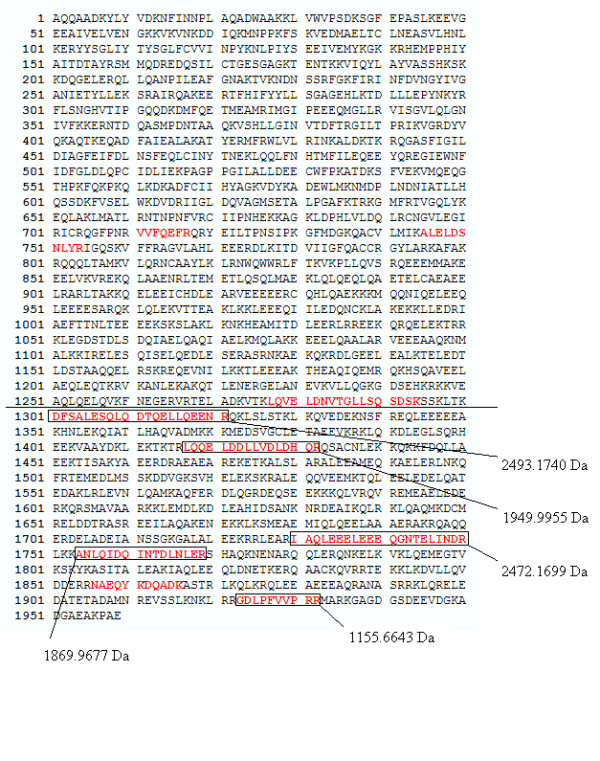
**Strong up-regulated peptides of Myosin-9 in the "glioma vessels group" versus the "normal vessels group" located at the C-terminus of the protein**. The strong up-regulated peptides are approximately located from amino-acid position 1301 to 1959.

From Table 5 it appears that peptides with mass 1869.9677, 1155.6643, 1949.9955, 2493.1740, and 2472.1699 of Myosin-9 are exclusively measured in the glioma vessels, while other peptides masses of Myosin-9 are more randomly distributed among the different groups, including normal vessels, glioma tissue, and normal tissue. Three peptides with different Wilcoxon-Mann-Whitney p-values, 0.00170, 0.04085, and 0.75200 are peptides of Myosin-9 identified by MS-MS sequencing (Additional file 1). Two of them are displayed in Figure 5 and 6. Figure 5 shows a fraction of the mass spectra of all samples, where the peak at peptide mass MH^+ ^of 1155.6643 Da of Myosin-9 displays an increased intensity in glioma samples represented by the dark lines, while the first isotopic mass of Neurofilament triplet L protein at 1154.7128 Da, represented by the grey lines, is as expected not present in glioma vessels. Neurofilament triplet L is identified by MS-MS sequencing in the "normal tissue and vessels" sample (see Additional file 1). Figure 6 shows an equal distribution of peak intensities among all samples of a Myosin-9 peptide mass at 1193.6166 Da. The strong up-regulated peptides of Myosin-9 are all located at the C-terminus of the protein, approximately from amino-acid position 1301 to 1959 (Figure 7). Table 7 shows the Wilcoxon-Mann-Whitney p-values of peptide-masses of Myosin-9 based on differences in peak intensities of each mass between the glioma vessels (series H) and normal vessels (series S) samples. The p-values are presented in the order of amino acid position of the peptide from the N-terminus of the protein. The p-value gradually drops from 0.60387 to 0.00310 from start positions 711 to 1923 of the amino acid sequences in the protein. The C-terminus of the tryptic fragment with mass 1155.6643 Da is amino acid sequence RR and known to be cut by trypsin at a lower rate. This explains the relative low peak intensity of 82*10^3 ^and 7*10^3 ^A.U. for glioma vessels and normal vessels, respectively (Table 7). By contrast, the p-value of peptides of fibronectin precursor in Table 5 remains constant. Some examples throughout different positions in the protein are a p-value of 0.02170 for the mass at 1401.6649 Da at start position 58, a p-value of 0.00503 for the mass at 1926.0516 Da at the middle position 1285, and a p-value of 0.02049 for the mass at 1818.9739 Da at the end position 2165 of the protein.

**Table 7:** Wilcoxon-Mann-Whitney p-values of Myosin-9 peptide peak mass intensities between the "glioma vessels" (series H) and "normal vessels" (series S) The p-value gradually drops from 0.60387 to 0.00310 from start position 711 to 1923 of the amino acid in the protein.

MS-MS	Position start	Position end	Observed mass MH^+^	Calculated mass MH^+^	ppm	Missed cleavages	Sequence	Wilcoxon-Mann-Whitney p-value	Mean intensity glioma vessels (H) *10^3 ^(A.U.)	Mean intensity normal vessels (S) *10^3 ^(A.U.)	Intensity H/S
	711	717	924.4946	924.49373	0.94	0	R.VVFQEFR.Q	0.60387	117	30	4
*	745	754	1193.6166	1193.61604	0.47	0	K.ALELDSNLYR.I	0.75200	100	44	2
	1277	1294	1946.0059	1946.00763	0.89	0	K.LQVELDNVTGLLSQSDSK.S	0.33559	287	179	2
*	1301	1321	2493.174	2493.17392	0.03	0	K.DFSALESQLQDTQELLQEENR.Q	0.04085	211	45	5
	1417	1432	1949.9955	1949.99268	1.45	0	R.LQQELDDLLVDLDHQR.Q	0.01285	264	95	3
	1730	1750	2472.1699	2472.17356	1.48	0	R.IAQLEEELEEEQGNTELINDR.L	0.05363	154	11	14
*	1754	1769	1869.9677	1869.96645	0.67	0	K.ANLQIDQINTDLNLER.S	0.00170	263	39	7
	1923	1932	1155.6643	1155.66325	0.91	1	R.GDLPFVVPRR.M	0.00310	82	7	11

* WARP-LC MS-MS MALDI-TOF glioma tissue and vessels (see additional file 1)

## Discussion

### Candidate glioma vessel formation biomarkers

In this study, proteins of laser micro-dissected tissues are analyzed using an improved high sensitivity detection, thus lower S/N threshold in comparison with our previous experiments [1]. Fibronectin precursor is 'rediscovered'. The mass 1275.5568 Da previously ascribed to acidic calponin [1] is measured again, however it differs relative much with 2.5 ppm from the calculated mass of 1275.5600 Da in this analysis. It is 5 times measured in the glioma vessels, 2 times in the normal vessels (including the outlier sample S10 in hierarchical clustering) and not in the other tissues. The mass 1659.8009 Da of Colligin-2 is measured again (Table 6) with 0.10 ppm mass accuracy. Cytoskeleton proteins and proteins involved in the calcium signaling pathway seem to be most up-regulated. Tubulin-specific chaperone A is likely to be detected by hierarchical clustering and reported to be biomarker for grade IV gliomas [19,20]. Annexin A5, detected with the Wilcoxon-Mann-Whitney test, is an ion channel protein with calcium- and phospholipid-binding properties. Calcium-binding protein p22 is a member of the calcium signaling pathway [21]. It is interesting that the mass of 1116.54323 Da was not identified in our previous study [1] matches with 0 ppm with a tryptic fragment of Calcium/calmodulin-dependent 3',5'-cyclic nucleotide phosphodiesterase 1C with SwissProt™ accession code Q14123, while the mass of 2157.1065 Da matches with 0.38 ppm with a tryptic fragment of Calcium/calmodulin-dependent protein kinase kinase 2 with SwissProt™ accession code Q96RR4. Both proteins belong to the same calcium signaling pathway [21]. The finding of only part of fragments of Myosin-9 up-regulated in the glioma vessel samples may have various reasons. It could be ascribed to splicing, fragmentation of Myosin-9 by proteases, or other technical and concentration related reasons. It is not unusual that some peptides or part of one protein may be up-regulated in one group. In a previous peptide-profiling study between a control and an end stage prostate cancer group (Figure 10 in ref. [2]), simultaneously up-regulated tryptic and down-regulated semi-tryptic peptide masses of one protein, namely human serum albumin affected by proteases were measured [22]. Some part of myosin-9 has a specific function since calponin binds (in addition to calmodulin) a specific region S2 of the Myosin-9 rod to actin [23]. Some relatively high p-values for fibronectin in Table 5 can be ascribed by wrongly clustered peptide masses of other proteins than fibronectin. Caldesmon binds Myosin-9 in the same S2 region. It is interesting that Glia maturation factor gamma (GMF-gamma) with SwissProt™ accession code O60234 is another protein preferentially expressed in human micro vascular endothelial cells that modulates actin cytoskeleton reorganization [24]. It could be measured in glioma vessels with one mass of 1762.8413 Da and a low Wilcoxon-Mann-Whitney p-value of 8.7 *10^-3^. Glia maturation factor gamma is measured co-expressed with CDC42 effector protein 3 in stromal vascular cells (see Table 3 in ref. [25]). It is suggested that Glia maturation factor gamma in combination with the protein CDC42 plays a role in angiogenesis [24].

### Mass accuracy

The theoretical mass accuracy is about 1.05 ± 0.27 ppm for the FTICR MS instrument used without centroiding. It decreases to 0.67 ± 0.48 ppm when determined on peptide masses, which appear to be presents in 18 samples or more in the peptide matrix of in total 38 samples. Probably, centroiding of the mean peptide mass takes place when taking the average over more samples. In a single high resolution MALDI-FTICR spectrum the real peak mass is between the data point with the local highest intensity and some of the data points with the next highest intensities. The measured peak mass of the data point with the highest local intensity is by change 50% left or right from the real peak maximum. It is hard to distinguish centroiding from effects of the dynamic range or ion-statistics. The mass accuracy decreases for peaks with low intensity. The peaks less frequently measured in all samples are probably also the less intensive which results in less accurate masses (Table 3). To limit performance problems a relative large peak search window of 20 ppm is chosen. The most abundant proteins have always the largest peak intensities within this window and the peptide masses of these proteins are chosen by the algorithm. There is more competition among the peptides of less abundant proteins with about equal low intensities. May be the combination of peptides from different proteins is the reason that the mass averaging does not improve accuracies by reducing statistical deviations for small peaks. The observation that the most intensive peaks do not have necessary the highest accuracy supports an additional effect of centroiding. A further improvement of the mass accuracy would significantly increase the reliability and direct identification of candidate proteins found using the MASCOT™ search engine on the SwissProt™ database. We applied a centroiding by a weighted mean calculated with various mass windows to 20 ppm in both directions of the peak mass, which is about the size of the peak-width, with a total window of 40 ppm. This did not improve the accuracy of the measured peptide masses, probably because the peaks are not symmetric. The larger number of peptide masses obtained by LC-MS demonstrates that there are a lot of overlapping peptide masses. This problem was recognized by Strittmatter et al. [26] who applied a double Gaussian fit to eliminate shoulders on the asymmetric peak distribution to get a more accurate mass. A double Gaussian fit is a special case of a Gaussian Mixture Modeling (GMM) [27-31], where 2 Gaussian curves are fitted through the peak distribution, with two maximum intensities at mass_1 _and mass_2 _separated by a distance Δ, thus mass_1 _– mass_2 _= Δ. We encounter the same failure of less likelihood of a convergence of masses, where mass_1 _= mass_2 _when applying a double Gaussian fit, but don't like to use the fixed Δ as suggested in [26].

### De-isotope algorithm

The introduced de-isotope algorithm, which only removes peaks which are isotopes in an initial window of 1.0034 ± 0.0100 Da leads to more peptide masses compared to the commercial software in the processed peak lists and consequently to more hits with calculated peptide masses of proteins in the SwissProt™ database by using the MASCOT™ search engine. Although we obtained satisfactory results with the de-isotope algorithm, the relative intensities of peaks within the isotopic clusters also should be compared with the theoretical distributions. Especially, the high masses display a larger scatter of isotopic distance and could easily fall outside the restrictive acceptance window (Figure 2). Ideally, it should be an (equipment dependent) function of mass. Since the isotopic distribution of individual elements, C, H, N, O and S of peptides is known, the abundance for each permutation of individual elements can be calculated with a multinomial distribution and the total number of atoms of the element in the peptide [32]. Combining the abundances for all the known number of different elements in a peptide, the isotopic distribution for a peptide can be calculated. Senko [13] calculates the number of each element C, H, N, O and S in a peptide by division of the peptide mass by the mass of a theoretical "averagine" amino acid, while Gay and co-workers [11] used all the tryptic fragments of the SwissProt™ database in a mass window. This resulted in a plot of the percentage intensity distribution of the 5 isotopes M^0^, M^1+^, M^2+^, M^3+^, M^4+^, and M^5+ ^as a function of the peptide mass in Da. Samuelsson and co-workers [12] have developed an algorithm to determine the mono-isotope masses in an overlapping cluster comparing the measured with the expected intensity distribution of the isotopic masses in a cluster. Valkenborg and co-workers [9] can distinguish peptides containing 0, 1 and 2 sulfur atoms from isotopic clusters, which would help with the analysis of proteins. The database application with such an algorithm can result in finding reliable mono-isotopic masses and perhaps leads to more protein identifications.

## Conclusion

A database application is presented that can compare hundreds of raw high resolution FTICR mass spectrometry files without serious performance limitations. A new less rigorous than commercial propriety software de-isotope algorithm is introduced, that results in more mono-isotopic peptide masses and consequently more protein identifications. From the peptide masses in the mass spectrometry files 2 peptide profile matrices are created taking the average of peptide masses in different samples within a mass window of 3 ppm and listing for the individual samples the 1) presence or absence of the peptide peaks and 2) peak intensities. The mass accuracy of the Java™ application is predominantly influenced by the data point resolution in the raw FTICR mass spectrometry files. Centroiding of peptide masses takes place by taking the average over more spectra in the profile matrix. The usage of raw MS spectra instead of peak lists results in a more reliable comparison of peak intensities between groups. The Wilcoxon-Mann-Whitney test can be performed on the intensity matrix, using raw spectrometry data. From this test it appears that cytoskeleton proteins and proteins involved in the calcium signaling pathway seem to be most up-regulated. Tryptic fragments at the C-terminus of the Myosin-9 protein are more up-regulated in glioma vessels compared to the peak intensities observed in normal vessels. The Wilcoxon-Mann-Whitney p-values of peptide fragments show a significant decline from the N-terminus of Myosin-9. The software described in this paper gives a new opportunity to find and quantify significantly differentially expressed peptides close to noise level in clinical samples.

## Abbreviations

ACQUS, Acquisition Status; AU, Arbitrary Units; CSV, Comma Separated Value; DHB, 2,5-DiHydroxyBenzoic acid; FFT, Fast Fourier Transformation; FID, Free Induction Decay; FTICR, Fourier Transform Ion Cyclotron Resonance; GB, Gaussian Broadening; GFAP, Glial Fibrillary Acidic Protein; GMM, Gaussian Mixture Modeling; GUI, Graphical User Interface; JAR, Java Archive; LB, Lorentzian Broadening; LC, Liquid Chromatography; LM, Linear Model; LPC, Laser Pressure Catapulting; MALDI, Matrix Assisted Laser Desorption Ionization; M/Z, Mass over Charge; MS, Mass Spectrometry; PPM, Parts Per Million, 10^-6^; TOF, Time of Flight; UPGMA, Un-weighted Pair Group Method with Arithmetic Mean

## Authors' contributions

MKT programmed and tested the Java code and GUI and R scripts, IS programmed the R routines, and MK helped with implementation of the Java^TM^ FFT module and reading the byte array format of the fid files. DANM prepared the micro dissected tissue samples. PCB did the MS analysis. JMK counterstained, and examined sections of 5 *μ*m of fresh-frozen samples of glioblastoma located in the cerebral hemispheres to verify the presence of proliferated tumor vessels. ACA, PAESS, and TML designed and wrote the research program. All authors read and agreed with the manuscript. TML and JMK contributed equally to this work

## Supplementary Material

Additional file 1 **MS-MS sequencing analysis results of the "glioma vessels", "normal vessels", "tissue surrounding the glioma vessels", and "tissue surrounding the normal vessels" samples**. A BTDX.xml file exported by the WARP-LC™ software, containing the MS and the MS-MS peak masses, is imported in Biotools™ 3.0 (build 1.68) (Bruker Daltonics, Germany) and submitted by this software application to the SwissProt™ version 40.21 database, using the MASCOT™ search engine, allowing 150 ppm parent mass tolerance, 0.5 Da fragments tolerance, and one missed trypsin cleavage site. Click here for file

Additional file 2 **Wilcoxon-Mann-Whitney p-values of the intensities of peak masses between the "glioma vessels" and "normal vessels" group**. The Wilcoxon-Mann-Whitney p-values are calculated based on intensity differences of peak masses between the "glioma vessels" and "normal vessels" group. The p-values are presented as well up-regulated (+) as down-regulated (-) in the "glioma vessels" group. Click here for file

Additional file 3 Peptide peak masses and intensities list exported from the database application with a signal to noise (S/N) ratio > 4. Click here for file

Additional file 4 Peptide peak masses and intensities list exported from the DataAnalysis™ 3.4 (Build 169) software with a signal to noise (S/N) ratio > 1.7. Click here for file

Additional file 5 Mono-isotopic peptide peak masses and intensities list exported from the DataAnalysis™ 3.4 (Build 169) software with the SNAP™ de-isotope algorithm and signal to noise (S/N) ratio > 1.7. Click here for file

Additional file 6 Mono-isotopic peptide peak masses and intensities list exported from the database application with the de-isotope algorithm described in this paper and a signal to noise (S/N) ratio > 4. Click here for file

Additional file 7 **Hierarchical clustering of the 38 of the in total 40 samples**. A number of 10 spectra of glioma blood vessels with codes H1 to H10, 10 spectra of tissue surrounding the glioma vessels with codes TH1 to TH10, 10 spectra of normal vessels with codes S1 to S10, and 10 spectra of tissue surrounding the normal vessels with codes TS1 to TS10 are included. The "normal vessel" sample S5 and "tissue surrounding the normal vessels" sample TS5 could not be internally calibrated and are not included. Click here for file

Additional file 8 Peptide profile matrix of present and absent masses in different samples generated by the Java™ application. Click here for file

Additional file 9 Installation instructions. Click here for file

Additional file 10 Create table script for the MySQL™ database. Click here for file

Additional file 11 Java Source code. Click here for file

Additional file 12 MALDI-FTICR MS test data. Click here for file
